# Combination Therapies to Inhibit the RAF/MEK/ERK Pathway in Melanoma: We are not Done Yet

**DOI:** 10.3389/fonc.2015.00161

**Published:** 2015-07-17

**Authors:** Grant A. McArthur

**Affiliations:** ^1^Department of Cancer Medicine, Peter MacCallum Cancer Centre, East Melbourne, VIC, Australia; ^2^Department of Pathology, University of Melbourne, Parkville, VIC, Australia; ^3^Department of Medicine, St Vincent’s Hospital, University of Melbourne, Fitzroy, VIC, Australia; ^4^Sir Peter MacCallum Department of Oncology, University of Melbourne, East Melbourne, VIC, Australia; ^5^Molecular Oncology Laboratory, Oncogenic Signaling and Growth Control Program, Peter MacCallum Cancer Centre, East Melbourne, VIC, Australia; ^6^Translational Research Laboratory, Cancer Therapeutics Program, Peter MacCallum Cancer Centre, East Melbourne, VIC, Australia

**Keywords:** BRAF, MEK, ERK, CDK4, apoptosis, BIM, melanoma

## The Combination of BRAF and MEK Inhibition in Advanced Melanoma

The discovery and development of small molecule inhibitors of mutant BRAF kinase and MEK kinase have revolutionized the care of patients with melanoma. When used as single agents, the BRAF inhibitors, vemurafenib and dabrafenib, improve progression free and overall survival when compared to chemotherapy with dacarbazine (1, 2). The MEK inhibitor, trametinib, also improves progression-free survival compared to chemotherapy, but achieves a lower response rate at its maximum tolerated dose in a continuous schedule when compared to BRAF inhibitors (3). Therefore, single agent trametinib is generally reserved for patients with intolerance to BRAF inhibitors, an uncommon event.

The identification of mechanisms of resistance to single agent BRAF inhibitors that reactivate the RAF/MEK/ERK pathway (4–6) led to the hypothesis that addition of a MEK inhibitor to a BRAF inhibitor may prevent or delay the emergence of resistance. This has indeed been the case. Three separate phase 3 trials have shown superiority of dual BRAF and MEK inhibition when compared to single agents BRAF inhibition alone (7–9). Dabrafenib combined with trametinib was superior to dabrafenib alone or vemurafenib alone, and vemurafenib and cobimetinib were superior to vemurafenib alone with hazard ratios for progression-free survival favoring the combination arms of 0.75, 0.56, and 0.51, respectively. Moreover, overall survival was also improved with hazard ratios, favoring the combination arms of 0.63, 0.69, and 0.65, respectively. Interesting combination therapy also substantially improved the complete response rate from 4–9 to 10–13% across the three studies.

In each of these three trials, it was also clear that combination therapy reduced the frequency of cutaneous side effects that have been attributed to BRAF inhibitor-induced “paradoxical activation” of RAF kinases in cells without BRAF mutations. This exactly predicts the findings from preclinical studies where MEK inhibition downstream of activated RAF kinases reduces signaling through ERK and the outputs of paradoxical activation (10). Overall, the combination of BRAF and MEK inhibition is remarkably well tolerated, alleviating the fear that combinations of signaling inhibitors, particularly those that target the same pathway, would not be tolerable. This is probably due to paradoxical activation of RAF kinases reducing the effects of MEK inhibition, and MEK inhibition reducing the effects of paradoxical activation of RAF kinases.

## Mechanism of Resistance to Combined BRAF and MEK Inhibition

Although the combined use of BRAF and MEK inhibition has clearly become a new standard for inhibiting the RAF/MEK/ERK pathway in patients with advanced BRAF mutant melanoma the problem of acquired resistance has become a major stumbling block to obtaining long-term disease control. In contrast, primary or innate resistance is very uncommon (7–9), suggesting that without the selective pressure of long-term pathway inhibition most melanoma cells with BRAF mutations are sensitive to RAF/MEK/ERK pathway inhibition.

A systematic approach using next generation sequencing of the exome of melanoma tissue from patients with acquired resistance has been employed by the Garraway, Lo, and Rizos groups to examine sequence variants of higher frequency in samples derived from lesions refractory to single agent BRAF inhibitors. Furthermore, RNA sequencing or reverse transcriptase-PCR has been used to examine the emergence of splice variants of BRAF that induce resistance to BRAF inhibition. With over 100 patient samples analyzed, a very clear pattern emerges with approximately two-thirds of patients acquiring genomic events that reactivate RAF/MEK/ERK signaling (5, 6, 11–16). This correlates well with immunohistochemistry and reverse phase protein array studies that show a similar fraction of samples from patients with resistance to BRAF inhibitors with elevated phosphorylation of ERK in samples consistent with reactivation of RAF/MEK/ERK signaling (17, 18). One-third of patients were predicted to have mechanisms that bypass the requirement for reactivation of RAF/MEK/ERK signaling.

Interestingly, the three most common molecular events reactivating RAF/MEK/ERK signaling, BRAF amplification, NRAS mutations, BRAF splice variants, all promote dimerization of mutant BRAF with CRAF or wild type BRAF. Both vemurafenib and dabrafenib inhibit mutant BRAF by binding to the ATP binding pocket that in the setting of mutant BRAF monomers potently inhibit RAF/MEK/ERK signaling. However, in the setting of upstream activation of RAS (e.g., mutant NRAS), or amplified BRAF or truncated mutant BRAF generated by the splice variant, paradoxical activation of the pathway is induced through generation of RAF dimers (19–22). Furthermore, even in the absence of these genomic variants, BRAF inhibitors can enhance upstream activation of RAS through feedback mechanisms, resulting in a new adapted steady state of active MEK and ERK (23–26). In all these settings, inhibition of MEK and/or ERK should reduce pathway output consistent with the clinical outcomes of patients treated with combined BRAF and MEK inhibition.

Given improved clinical outcomes of combined BRAF and MEK inhibition and frequent reactivation of RAF/MEK/ERK signaling as a mechanism of resistance to BRAF inhibitor monotherapy, there is immense interest in understanding the mechanisms of resistance to combined BRAF and MEK inhibition. Because the combination of BRAF and MEK inhibition may more effectively inhibit RAF/MEK/ERK signaling one may predict *a priori* two possible outcomes: first, with more effective inhibition of RAF/MEK/ERK signaling, there would be an increase in the frequency of bypass mechanisms independent of RAF/MEK/ERK signaling given a higher threshold to reactivate RAF/MEK/ERK signaling; or second, with more effective inhibition of RAF/MEK/ERK signaling, it would be advantageous, or even essential to reactivate RAF/MEK/ERK signaling by further genomic events for cell proliferation to occur.

Studies from the Garraway, Lo, Rizos, and Chin groups clearly show the acquisition of sequence variants activating RAF/MEK/ERK signaling in patients developing resistance to combined BRAF and MEK inhibition (18, 27–29). These genomic events again include BRAF amplification, NRAS mutations, BRAF splice variants, and MEK mutations. If such studies are confirmed on larger datasets, this sets up the intriguing concept that as the RAF/MEK/ERK pathway is inhibited more effectively it becomes essential for a cell to overcome this inhibition if proliferation is to occur. Therefore, it follows that if the pathway can be even more effectively inhibited one may be able to raise the threshold for genomic events to reactivate the pathway so high that the frequency of acquired resistance could be dramatically reduced.

## Consequences of More Effective Inhibition of the RAF/MEK/ERK Pathway

Inhibition of RAF/MEK/ERK signaling in melanoma cells with BRAF mutations results in cell cycle arrest and promotion of cell death, including apoptosis. Clinically, this manifests in reduced size of tumor masses, which is partial or even complete response. In support of this concept, there was a correlation between inhibition of phosphorylation or ERK and reduction in tumor size in patients treated with vemurafenib (30). Moreover, as mentioned above, combined BRAF and MEK inhibition increase the frequency of complete responses. However, it is worth considering the consequences of pathway inhibition in more detail. First, pathway inhibition can result in cells adapting to the inhibition of signaling with the acquisition of mesenchymal phenotype with enhanced cell migratory capacity and a change in cell metabolism (31–34). This allows cells to survive and potentially enables subsequent outgrowth of resistant cells. Second, the tumor microenvironment must change with therapy. There is a change in the leukocytic content of tumors (35–37), tumors contain dead and dying cells and some cells may acquire senescence-like features (38). All these factors may influence whether a cell capable of generating acquired resistance survives, dies, or is enforced into a non-proliferative state that maybe long term.

**Figure 1:**
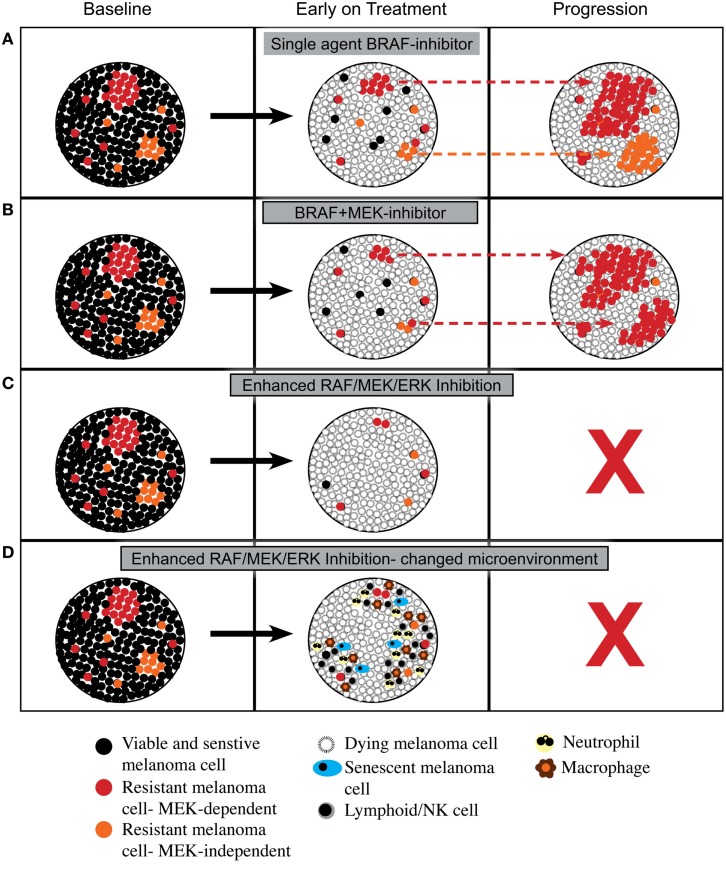
**Proposed cellular responses to inhibition of RAF/MEK/ERK signaling**. **(A)** Response to single agent BRAF inhibitor with induction of cell death and then out growth of resistant cells having RAF/MEK/ERK-dependent mechanisms of resistance or RAF/MEK/ERK-independent mechanisms of resistance. **(B)** Response to combined BRAF and MEK inhibitors with induction of cell death and then out growth of resistant cells dominated by RAF/MEK/ERK-dependent mechanisms of resistance. **(C)** Response to enhanced inhibition of RAF/MEK/ERK signaling with induction of greater cell death leading to tumor load being below a critical threshold required for outgrowth of resistant cells. **(D)** Response to enhanced inhibition of RAF/MEK/ERK signaling with induction of greater cell death plus a change in tumor microenvironment with influx of leukocytes that prevents emergence of resistance.

As summarized in Figure 1, enhanced inhibition of the RAF/MEK/ERK pathway may lead to more cell death or even a change in tumor microenvironment that is less compatible with long-term cell survival or the reacquisition of a proliferative state. This hypothesis remains speculative; however, the increased proportion of patients achieving complete response with combined BRAF and MEK inhibition, and the excellent survival of patients who achieve a complete metabolic response on FDG-PET scan (39), that is, a surrogate of inhibition of the RAF/MEK/ERK pathway (40), suggest that more effective or complete inhibition of RAF/MEK/ERK signaling may indeed produce biological responses that improve overall survival.

## Strategies to Enhance Inhibition of the RAF/MEK/ERK Pathway

There are a number of strategies that might improve inhibition of the RAF/MEK/ERK pathway beyond that obtained with continuous exposure to BRAF and MEK inhibitors. Dose, schedule, potency, and inhibiting ERK all have the potential to reduce output from the pathway and result in improved clinical outcomes. A second approach is to inhibit key components of the pathway downstream of ERK. This includes CDK4, pro-apoptotic molecules, such as BIM, and even other signaling networks vital to the outputs of the pathway.

It has become a “tradition” to inhibit oncogenic signaling continuously following the early success of this approach in targeting BCR-ABL with imatinib (41). However, preclinical data suggest that intermittent therapy can allow an increase in dose and greater inhibition of oncogenic signaling when targeting BCR-ABL (42) or BRAF (43). Moreover, intermittent therapy allows reversal of cell adaptation referred to above (32), potentially re-sensitizing cells that survive pathway inhibition to reintroduction of the inhibitors. Interestingly, withdrawal of pathway inhibition may also lead to heightened ERK activity as a “rebound response” leading to tumor regression (29, 43). This approach of intermittent therapy targeting BRAF in melanoma has been partially examined by the use of a schedule of 3 weeks on and 1 week off of the MEK inhibitor cobimetinib when combined with the BRAF inhibitor vemurafenib that is given continuously (44). The intermittent schedule allows a higher dose of cobimetinib to be delivered with likely greater inhibition of pathway output for 3 weeks out of 4 on cobimetinib and vemurafenib when compared to 1 week out of 4 on vemurafenib alone. However, more prolonged interruption of pathway inhibition may provide greater benefits and is being investigated in a clinical trial randomizing patients with advanced BRAF mutant melanoma to continuous or intermittent exposure to dabrafenib and trametinib (NCT02196181).

It is also possible to inhibit the output of the pathway by more effectively inhibiting MEK or possibly by targeting ERK. In the case of trametinib, this can occur through reduction of CRAF/MEK complexes (45). Trametinib, the MEK inhibitor, currently approved in melanoma along with other MEK inhibitors in clinical development are allosteric inhibitors (45, 46). In contrast, the ERK inhibitors in clinical development target the ATP binding pocket of the kinase. These properties may influence the output of the pathway when the agents are used in combination possibly due to suppression of feedback mechanisms when compared with MEK inhibitors. Indeed, different allosteric MEK inhibitors can affect feedback to MEK inhibition (45–47) and similar differences may also occur with ERK inhibitors. Furthermore, covalent irreversible inhibitors of ERK have been developed that may further differentiate these agents from MEK inhibitors (48), (NCT02313012). So, further preclinical and clinical data with ERK inhibitors and novel MEK inhibitors are warranted.

A number of approaches can be taken to inhibit the RAF/MEK/ERK pathway downstream of ERK. It is clear that CDK4 activation by ERK is critical to the ability of RAS or RAF to promote cell cycle progression (49). Moreover, genomic changes in the CDK4 regulatory network affect outcomes in patients treated with BRAF inhibitors and can induce resistance (15, 50). Interesting CDK4 inhibition can induce irreversible cell cycle arrest and senescence in melanoma cells with BRAF mutations (38). Therefore, combining CDK4 inhibitors with inhibitors of RAF, MEK, and/or ERK is a promising approach that is actively being pursued preclinically and clinically (51).

Inhibition of the RAF/MEK/ERK pathway can induce apoptosis, principally through activation of the BH3 alone protein BIM (52, 53), mitochondrial relocalization of BMF (54), and reduction of the anti-apoptotic molecule MCL1 (52). Therefore, the possibility of selectively enhancing the pro-apoptotic affects of RAF/MEK/ERK pathway inhibition through the use of BH3-mimetics in combination with pathway inhibitors is being investigated in clinical trials (NCT01989585).

Finally, the understanding that ERK can influence other signaling networks offers additional strategies to enhance the biological outcomes of ERK inhibition. Interestingly, protein translation can be regulated by ERK with signaling converging on the EIF4E/4G complex (55) that may also be influenced by the mTOR pathway (56). Therefore, one approach to enhance inhibition of the RAF/MEK/ERK pathway is to combine inhibitors of protein translation or inhibitors of mTORC complexes with BRAF and/or MEK inhibitors.

## Conclusion

The rationale for ongoing investigation of therapeutic strategies to enhance inhibition of the RAF/MEK/ERK pathway is strong. Moreover, the impact of pathway inhibition in the adjuvant setting where there maybe differences in the extent of response of the micro-metastases, or in the microenvironment that emerges following treatment with BRAF and MEK inhibitors necessitates ongoing preclinical and clinical research into therapeutic targeting of the pathway. While it is clear that combination approaches that look at simultaneous or sequential use of immunotherapeutic approaches with agents that target the RAF/MEK/ERK pathway are also a priority, it is not time to divert attention away from the pathway that induces such profound oncogene addiction in melanoma patients whose tumors contain activating mutations in BRAF.

## Conflict of Interest Statement

Prof. Grant A. McArthur is a compensated consultant for Provectus and an uncompensated consultant for Bristol-Myers Squibb, GlaxoSmithKline, Amgen, Novartis, and Roche-Genentech, and receives research funding from Pfizer, Celgene, and Ventana.
